# Enzyme Activity Profiles Produced on Wood and Straw by Four Fungi of Different Decay Strategies

**DOI:** 10.3390/microorganisms8010073

**Published:** 2020-01-02

**Authors:** Eliana Veloz Villavicencio, Tuulia Mali, Hans K. Mattila, Taina Lundell

**Affiliations:** Department of Microbiology, Faculty of Agriculture and Forestry, Viikki Campus, University of Helsinki, FI-00014 Helsinki, Finland; eliana.veloz-villavicencio@helsinki.fi (E.V.V.); tuulia.mali@helsinki.fi (T.M.); hans.mattila@helsinki.fi (H.K.M.)

**Keywords:** *Basidiomycota*, wood decay fungi, lignocellulose, biodegradation, carbohydrate active enzymes, laccase, manganese peroxidase, enzyme activity assays, organic acids, oxalic acid

## Abstract

Four well-studied saprotrophic *Basidiomycota Agaricomycetes* species with different decay strategies were cultivated on solid lignocellulose substrates to compare their extracellular decomposing carbohydrate-active and lignin-attacking enzyme production profiles. Two *Polyporales* species, the white rot fungus *Phlebia radiata* and brown rot fungus *Fomitopsis pinicola*, as well as one *Agaricales* species, the intermediate “grey” rot fungus *Schizophyllum commune*, were cultivated on birch wood pieces for 12 weeks, whereas the second *Agaricales* species, the litter-decomposing fungus *Coprinopsis cinerea* was cultivated on barley straw for 6 weeks under laboratory conditions. During 3 months of growth on birch wood, only the white rot fungus *P. radiata* produced high laccase and MnP activities. The brown rot fungus *F. pinicola* demonstrated notable production of xylanase activity up to 43 nkat/mL on birch wood, together with moderate β-glucosidase and endoglucanase cellulolytic activities. The intermediate rot fungus *S. commune* was the strongest producer of β-glucosidase with activities up to 54 nkat/mL, and a notable producer of xylanase activity, even up to 620 nkat/mL, on birch wood. Low lignin-attacking but moderate activities against cellulose and hemicellulose were observed with the litter-decomposer *C. cinerea* on barley straw. Overall, our results imply that plant cell wall decomposition ability of taxonomically and ecologically divergent fungi is in line with their enzymatic decay strategy, which is fundamental in understanding their physiology and potential for biotechnological applications.

## 1. Introduction

Lignocellulose is one of the most abundant renewable resources on Earth [1,2]. Decomposition of the lignocellulosic plant biomass materials in nature is carried out by microorganisms. Filamentous saprotrophic fungi of *Basidiomycota* play an important role in the cycling of carbon and other elements due to their capacity to convert the complex biopolymeric carbohydrate components of the compact plant cell wall lignocelluloses into sugars and monomeric compounds [1,2,3,4,5].

Fungi of *Basidiomycota*, with emphasis on species of the taxonomic class *Agaricomycetes*, possess multi-enzyme encoding gene sets that make them capable of decomposing complex substrates such as the carbohydrate polysaccharides (cellulose and hemicelluloses) and lignin moieties embedded in lignocelluloses [1,3,4,5,6,7,8,9]. According to the type of decay and growth on solid wood or grass plant residues, saprotrophic *Basidiomycota Agaricomycetes* species are grouped with white rot fungi, brown rot fungi and soil-inhabiting litter-decomposing fungi, together with ectomycorrhizal and tree plant-pathogenic fungi [1,4,5,8,9,10]. In the *Agaricomycetes* order *Polyporales*, species of white and brown rot decay trait are dominant together with a few ectomycorrhizal symbionts, whereas in the diverse order *Agaricales*, lifestyles from ectomycorrhizal to grass litter-decomposing and intermediate wood-decay types are found [4,6,8,9,11].

White rot fungi are well-known decomposers of wood lignin, while they depolymerize and utilize cellulose and hemicellulose for heterotrophic growth [1,2,3,4,5,6,7,8,9,12]. White rot fungi possess an extensive set of carbohydrate-active enzymes (CAZymes) [13] that act on cellulose, hemicellulose and pectin, as well as lignin-modifying enzymes (AA2 family of class-II heme including peroxidases) to oxidize and break down the complex lignin polymers [14]. These class-II peroxidases (lignin peroxidases (LiPs), versatile peroxidases (VPs), and manganese peroxidases (MnPs)), together with other auxiliary CAZyme oxidoreductase enzymes, generate oxidative reactions affecting and degrading lignin moieties in wood [3,4,6,13,14]. Laccases, in turn, are generally expressed and secreted by *Agaricomycetes* fungi and found in multigene families in their genomes [1,4,5,8,10,12,15]. Together with class-II peroxidases, laccases may participate in oxidation of lignin, in combination with low-molecular-weight phenolics and nitrogen-substituted compounds [2,3,5,15,16].

*Agaricomycetes* brown rot fungi efficiently depolymerize the lignocellulose polysaccharides, cellulose and hemicellulose, whereas lignin moieties are only slightly modified leaving behind a dry brownish residue [1,8,10,16]. In contrast to white rot fungi, brown rot fungi have lost their class-II peroxidase-encoding genes, and depolymerization of plant cell wall polysaccharides proceeds mainly via non-enzymatic Fenton reactions generated outside the hyphae [16,17]. Breakdown processes are enhanced by the production of cellulolytic and hemicellulolytic activities of glycoside hydrolase CAZymes of e.g., families GH3, GH5 and GH10 [10,11,16,17].

Litter-decomposing *Agaricomycetes* fungi of the order *Agaricales* generally inhabit soil environments including plant residual litter such as pieces of wood, branches, needles, and leaves deposited on the forest floor [1]. A few species of *Agaricales* such as *Schizophyllum commune* are wood-colonizers with various decay strategies. *Agaricales* fungi also secrete an array of CAZymes, especially against plant cell wall polysaccharides, and some species (e.g., of the genera *Agaricus*, *Agrocybe* and *Pleurotus*) have the ability to produce lignin-modifying and other peroxidases, thus resembling white rot fungi in their decomposition strategy [4,9,14,18,19]. The litter-decomposing fungus *Coprinopsis cinerea* secretes various CAZymes, and laccases and variant peroxidases apparently for detoxification and activation of soil humic substances [14]. Thus, both litter-decomposers and wood-decaying *Agaricomycetes* fungi play an important role in recycling of carbon and other nutrients especially in the forest ecosystems.

In this study, extracellular enzyme profiles of four common saprotrophic *Basidiomycota Agaricomycetes* species from two taxonomic orders (*Polyporales* and *Agaricales*) with different lifestyles and decay strategies were compared by cultivating the fungi in solid-state cultures on birch wood or barley straw. The main aim of the study was to record specific enzyme production patterns of the fungi to explain their eco-physiological differences and substrate specificities. Also, effective producers of CAZYmes were examined, aiming at the applicability of these fungi in biotechnological processes, such as lignocellulose pretreatments, waste bioconversions, and production of biofuels. In this respect, especially the high xylanase and β-glucosidase activities produced by *S. commune*, and laccase and MnP activities produced by *P. radiata*, are promising.

## 2. Materials and Methods

### 2.1. Fungal Isolates

The *Basidiomycota* isolates of the study are stored in the Fungal Biotechnology Culture Collection of the Microbial Domain Biological Resource Centre HAMBI (HAMBI mBRC, Helsinki, Finland), which is a part of the Biodiversity Collections Research Infrastructure in the Helsinki Institute of Life Science, University of Helsinki. The fungi belong to the taxonomic class *Agaricomycetes* order *Polyporales* (*Phlebia radiata* and *Fomitopsis pinicola*) and order *Agaricales* (*Schizophyllum commune* and *Coprinopsis cinerea*) and have their distinct decay strategies and lignocellulose substrate specificities (Table 1). *S. commune* H4-8 homokaryon [20] was obtained from H. Wösten, and *C. cinerea Amut Bmut* homokaryon [21] from L. Nagy, whereas the *P. radiata* and *F. pinicola* wild-type isolates originate from Finland [12,22]. Fungi were first cultivated on 2% (*w*/*v*) malt extract (Biokar Diagnostics, France) agar (MEA) plates at 25 °C in the dark for 7 days. The species have been genome-sequenced with annotated genomes available at the DOE Joint Genome Institute (JGI) MycoCosm genome repository [23].

### 2.2. Cultivation and Sampling

The wood-decaying fungi *P. radiata*, *F. pinicola* and *S. commune* were cultivated on small pieces of silver birch (*Betula pendula*) deciduous wood according to their substrate preferences (Table 1). Wood originated from a single tree felled in spring 2018 in South of Finland, cut to pieces, dried at room temperature, and sieved with a 5 mm mesh size metal sieve to obtain homogenous particle size distribution of the lignocellulose substrate. For *C. cinerea*, which is a non-wood-decaying but litter-decomposing fungus (Table 1), air-dried pieces (length about 2 cm) of barley (*Hordeum vulgare*) straw obtained from the 2017 autumn harvest of the University of Helsinki Viikki Campus research crop fields were used as substrate.

Pieces of wood and cut barley straw were oven-dried at 60 °C for 7 days and at 70 °C for 3 days, respectively. Three grams (in dry weight) of either wood or straw were weighed into each 100 mL conical glass flask closed with a cellulose stopper and a metal cap, dry run autoclaved (121 °C, 15 min), and supplemented with 10 mL of 0.5 % (*w*/*v*) yeast extract solution in Milli-Q water (autoclaved at 121 °C, 15 min before use) for availability of nitrogen, vitamins and trace elements for hyphal growth. Solid-state lignocellulose cultivations were inoculated with placing one ca. 0.5 cm^2^ sized mycelial plug of pre-cultured MEA on top of the solid lignocellulose in each 100 mL flask. In addition, cultivations on 2% (*w*/*v*) malt extract liquid (ME medium, pH 5.5) were started by inserting one mycelial plug into 50 mL of pre-autoclaved medium in a 250 mL conical flask and closed with cellulose stoppers. Three parallel culture flasks on both media were performed for each fungus, then incubated in the dark at 25 °C without agitation for 4 weeks (liquid ME cultivations) and 4 weeks (barley straw) to 12 weeks (birch wood) cultures.

Samples (1 mL) for enzyme assays were aseptically taken once a week from the liquid phase of the cultures, and stored at −20 °C in plastic sample tubes. Sterile MilliQ-water (1 mL at first week, 2 mL during weeks 2–12) was added into the lignocellulose cultures to keep the moisture and water content stable during the cultivation. For controls, flasks excluding fungi were incubated under the same conditions for both lignocellulose substrates (five to nine weeks) and ME medium (four weeks). All cultivations were performed with three biological replicates (three parallel culture flasks) and the mean value of three biological replicate flasks is reported in the results.

### 2.3. Enzyme Activity Assays

Samples were quickly thawed, vortexed and centrifuged (13,000× *g*, 4 °C, 5 min) before enzyme activity measurements. In the 96-well plate assays, each sample was repeated in three reactions (individual wells) as technical reaction replicates. Enzyme activities are reported as mean values and standard deviation of the three samples taken from the biological replicate cultivation flasks. Laccase, manganese peroxidase (MnP), β-glucosidase, endoglucanase, and xylanase activities were determined using 96-well plate methods previously established [7,22,24] using Spark multimode microplate reader (Tecan, Männedorf, Switzerland). Polypropylene microwell 96-well plates were used to measure laccase, β-glucosidase, endoglucanase, and xylanase activities in a final volume of 250 μL. For MnP activity measurements, UV-transparent microplates were used (Corning Inc., Corning, NY, USA). For the samples of *F. pinicola*, laccase activity was measured for the first and last three time points only, and MnP activity measurement was excluded due to the lack of class-II peroxidase genes in the species genome [4,6] and our previous experiences with the same isolate [17,22].

For laccase and MnP activities, ABTS (2,2’-azino-bis(3-ethylbenzothiazoline-6-sulphonic acid) and Mn^2+^ ions coupled with the addition of H_2_O_2_ were used as substrates, respectively, in 50 mM sodium malonate buffer (pH 4.5) [24]. For β-glucosidase activity, 1 mM of 4-nitrophenyl β-D-glucopyranoside was used as a substrate, whereas endoglucanase and xylanase activities were determined by using the coupled dinitrosalicylic acid (DNS) method with 1 % (*w*/*v*) hydroxyethyl cellulose and beech wood xylan, respectively, as substrates [7]. In the two latter assays, samples from the ME liquid cultures showed high background sugar content and interference in reactions with DNS. Therefore, net estimated activity was calculated by subtracting the background absorbance of the liquid medium control without fungus from the sample absorbance values.

### 2.4. Fungal-Produced Organic Acids and Culture Acidity

Organic acids produced by fungi cultivated on the solid-state lignocellulose substrates were quantified by adopting the UHPLC method previously optimized [25]. Supernatants of the centrifuged samples were treated by filtering 200 µL volume through 0.2-μm pore-size membrane GE Healthcare Whatman syringe filters (Thermo Fisher Scientific, Waltham, MA, USA) to remove disturbing agglomerates, into brown chromatography sample vials with integrated glass insert (Chromacol, Thermo Fisher Scientific, Waltham, MA, USA). If necessary, the samples were stored in their vials at −20 °C not more than 2 weeks prior to analysis by Agilent 1290 Infinity binary LC 171 system (Agilent Technologies, Santa Clara, CA, USA) coupled with a guard cartridge (Agilent Technologies) and reverse-phase Luna C18 column (150 mm by 4.6 mm, 3 μm particle size; Phenomenex, Torrance, CA, USA). Injection volume was 2 µL, column temperature 40 °C, and separation was performed at a flow rate of 0.950 mL min^−1^ under isocratic conditions by using an eluent mixture of 95 % (*v*/*v*) 0.3 % H_3_PO_4_ in Milli-Q water and 5 % (*v*/*v*) acetonitrile. Elution of carboxylic acids was followed as absorbance at 210 nm. For identification and quantitation, sets of commercially available reference organic acids were run with the same method [25]. The amounts of organic acids produced are presented as mean values with a standard deviation of the three biological replicate cultures.

The generation of acidity in the cultivations was followed each week by measuring the pH values of the centrifuged liquid samples at room temperature using an Orion 920A pH-meter equipped with a glass pH combinatory electrode (Thermo Fisher Scientific, Waltham, MA, USA). Results are presented as mean values with a standard deviation of the three biological replicate flask cultures.

### 2.5. Statistical Analyses

Statistical significance of the changes in the enzyme activity values observed and calculated at the cultivation time points was estimated using a one-way repeated measures ANOVA with post hoc Bonferroni correction. To estimate the statistical significance between the highest enzyme activity values obtained per fungus on the lignocellulose substrate and in the liquid ME medium, Student’s *t*-test was applied with Levene’s test for the homogeneity of each data set. A significance of *p* value ≤ 0.05 (confidence level over 95 %) is indicated for the results. ANOVA and *t*-test were applied using IBM SPSS Statistics 25 software package (Armonk, NY, USA).

## 3. Results

### 3.1. Laccase Activity

**Figure 1:**
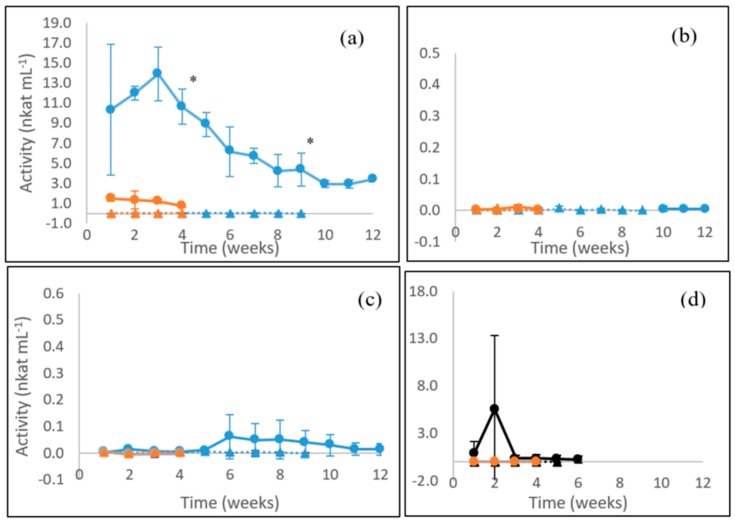
Laccase activities produced by (**a**) the WR fungus *P. radiata*; (**b**) the BR fungus *F. pinicola*; (**c**) the GR fungus *S. commune*; and (**d**) the LDF species *C. cinerea*. Fungal cultivations on birch wood (blue line with spheres), on barley straw (black line with spheres) and on liquid ME medium (orange line with spheres). Values from control incubations of birch wood (blue triangles), barley straw (black triangles) and liquid ME medium (orange triangles) without fungi are included. Mean value (*n* = 3) is presented at each week time point. Bars indicate the standard error. * statistically significant changes between time points. Notice different scales in the y axis. For information on the fungi, see Table 1.

Laccase activities were the highest in cultures of the white rot fungus *P. radiata* on the solid-state lignocellulose substrate (on birch wood, Figure 1a) and measurable during the 12 weeks of growth. However, for the other three species, negligible (*F. pinicola*) to very low levels (*S. commune*) or transient production (*C. cinerea*) of laccase was observed (Figure 1b–d). *P. radiata* laccase activity reached its maximum point on week three (13.9 nkat mL^−1^), then significantly (*p* ≤ 0.05) decreasing until cultivation at week nine to a stable level (4.4 nkat mL^−1^) (Figure 1a). The high standard deviation observed on the first week in laccase activities on birch wood (Figure 1a) indicated that the production of laccase by *P. radiata* was not synchronous between the three replicate culture flasks at the beginning. From cultivation week two onwards, the laccase activities were more homogeneous between the replicate flasks. On liquid ME medium, however, production of laccase by *P. radiata* during the 4 weeks of cultivation was very low, with maximal activity level of 1.5 nkat mL^−1^ reached at the first time point (Figure 1a).

### 3.2. MnP Activity

**Figure 2:**
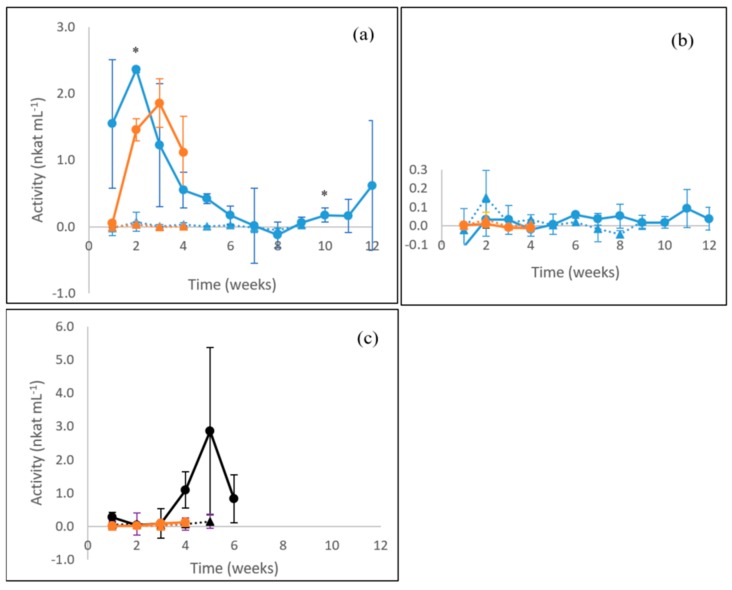
Manganese peroxidase (MnP) activities produced by (**a**) the WR fungus *P. radiata*; (**b**) the GR fungus *S. commune*; and (**c**) the LDF species *C. cinerea*. Fungal cultivations on birch wood (blue line with spheres), barley straw (black line with spheres) and liquid ME medium (orange line with spheres). Values from control incubations of birch wood (blue triangles), barley straw (black triangles) and liquid ME medium (orange triangles) without fungi are included. Mean value (*n* = 3) is presented at each week time point. Bars indicate the standard error. * statistically significant value between the time points. For information on the fungi, see Table 1.

The white rot fungus *P. radiata* showed a peak of MnP activity (up to 2.4 nkat mL^−1^) on the second week of cultivation on birch wood (Figure 2a), after which the activity significantly decreased (*p* ≤ 0.05) until week ten. On the liquid ME medium, *P. radiata* MnP activity peaked similarly, early on cultivation week three (1.9 nkat mL^−1^). On the contrary, no MnP activity was observed in the cultivations of the “grey rot” fungus *S. commune* (Figure 2b). Regarding *C. cinerea*, the MnP activity observed was low during the first weeks of cultivation on barley straw but then rapidly increased within 2 weeks (up to 2.9 nkat mL^−1^) (Figure 2c). However, the activity peak on week five is not completely reliable due to high deviation of the parallel samples observed as standard error. No production of MnP activity was detected on liquid ME medium for *S. commune* or *C. cinerea*.

### 3.3. β-glucosidase Activity

**Figure 3:**
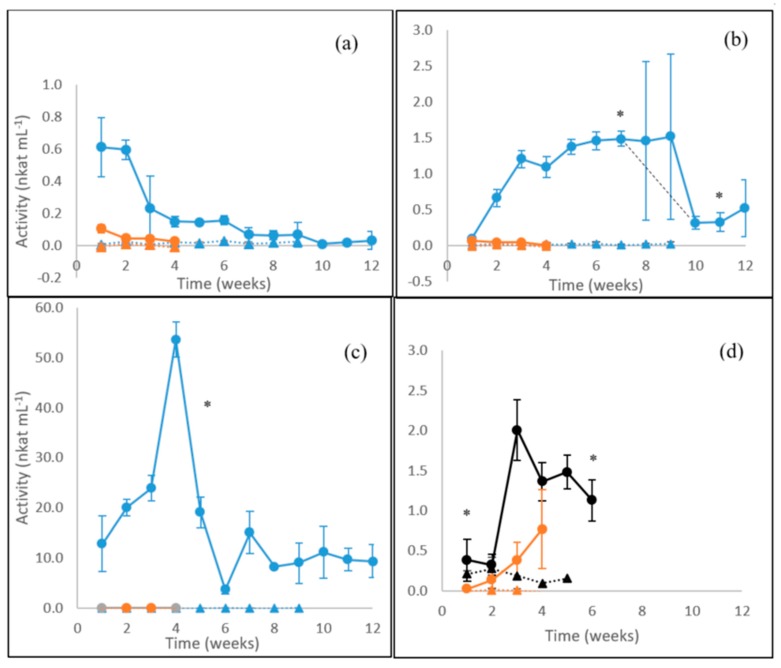
β-glucosidase activities produced by (**a**) the WR fungus *P. radiata*; (**b**) the BR fungus *F. pinicola*; (**c**) the GR fungus *S. commune*; and (**d**) the LDF species *C. cinerea*. Fungal cultivations on birch wood (blue line with spheres), on barley straw (black line with spheres) and on liquid ME medium (orange line with spheres). Values from control incubations of birch wood (blue triangles), barley straw (black triangles) and liquid ME medium (orange triangles) without fungi are included. Mean value (*n* = 3) is presented at each week time point. Bars indicate the standard error. * statistically significant changes between time points. Notice different scales in the y axis. For information on the fungi, see Table 1.

Activities of β-glucosidase lower than 1 nkat mL^−1^ were observed for the white rot fungus *P. radiata* on birch wood with the highest activity levels produced during the first 2 weeks of cultivation followed by a decline (Figure 3a). For the brown rot fungus *F. pinicola*, an opposite profile of β-glucosidase activity was observed with an increase during the first 3 weeks up to significant (*p* ≤ 0.05) steady levels of activity (1.5 nkat mL^−1^) on birch wood observable until cultivation week 7 followed by a rapid decrease to 0.3 nkat mL^−1^ on week ten (Figure 3b). The dashed black line in Figure 3b indicates this drop, which presented high standard deviation (high error bars) between the three parallel culture flasks. Only negligible β-glucosidase activities were observed on the liquid ME medium for *P. radiata* or *F. pinicola*. (Figure 3a,b).

The highest β-glucosidase activity was observed for *S. commune* on birch wood (Figure 3c), reaching a notable maximum (54 nkat mL^−1^) on week four, then significantly (*p* ≤ 0.05) decreasing to a steady level (9.4 nkat mL^−1^) until the end of cultivation. In contrast, no β-glucosidase activity was observed in the liquid ME medium within the 4 weeks of cultivation. In barley straw cultures of the litter-decomposing fungus *C. cinerea*, β-glucosidase activity significantly (*p* ≤ 0.05) increased from low levels of 0.4 nkat mL^−1^ during the third cultivation week and staying near to 1.5 nkat mL^−1^ until the sixth week (Figure 3d). In the liquid ME cultures, this fungus was the only producer of β-glucosidase activity with an increasing tendency during the 4 weeks of cultivation.

### 3.4. Endoglucanase Activity

**Figure 4:**
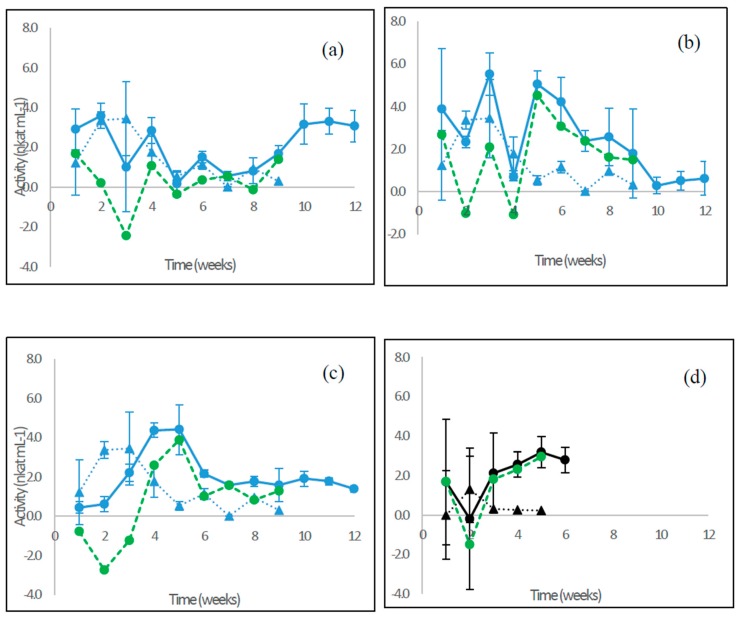
Endoglucanase activities produced by (**a**) the WR fungus *P. radiata*; (**b**) the BR fungus *F. pinicola*; (**c**) the GR fungus *S. commune*; and (**d**) the LDF species *C. cinerea*. Fungal cultivations on birch wood (blue line with spheres) and on barley straw (black line with spheres). Values from control incubations of birch wood (blue triangles) and barley straw (black triangles) without fungi are included. Net activity values are also shown (green dotted lines) after subtracting the control activity. Mean value (*n* = 3) is presented at each week time point. Bars indicate the standard error. Notice different scales in the y axis. For information on the fungi, see Table 1.

Low levels of endoglucanase activities around 4 nkat mL^−1^ were observed in the birch wood cultivations with *P. radiata*, *F. pinicola*, and *S. commune*, as well as for *C. cinerea* on barley straw (Figure 4). Activities fluctuated from the beginning until the end of the cultivations.

For the WR fungus *P. radiata*, the net endoglucanase activity calculated on birch wood was below 2.0 nkat ml^−1^ and fluctuating during the cultivation period (Figure 4a, green line). Similar to *P. radiata*, the net estimated endoglucanase activity in birch wood cultures of the brown rot fungus *F. pinicola* was at first fluctuating (Figure 4b, green line) and then peaked from week five (4.5 nkat mL^−1^) onwards; endoglucanase activity was measurable until the last weeks of cultivation. The “GR” fungus *S. commune* in turn produced endoglucanase activity distinctively on birch wood starting from week four, with the peak of activity reached on week five (3.9 nkat mL^−1^) and then continuing until the end of cultivation at levels lower than 2.0 nkat mL^−1^ (Figure 4c). On barley straw cultures of the LDF *C. cinerea*, net endoglucanase activity was observed since the beginning of the cultivation, and after the drop on week two, increasing activity up to about 3 nkat mL^−1^ was observed until the end of cultivation on week six (Figure 4d).

### 3.5. Xylanase Activity

**Figure 5:**
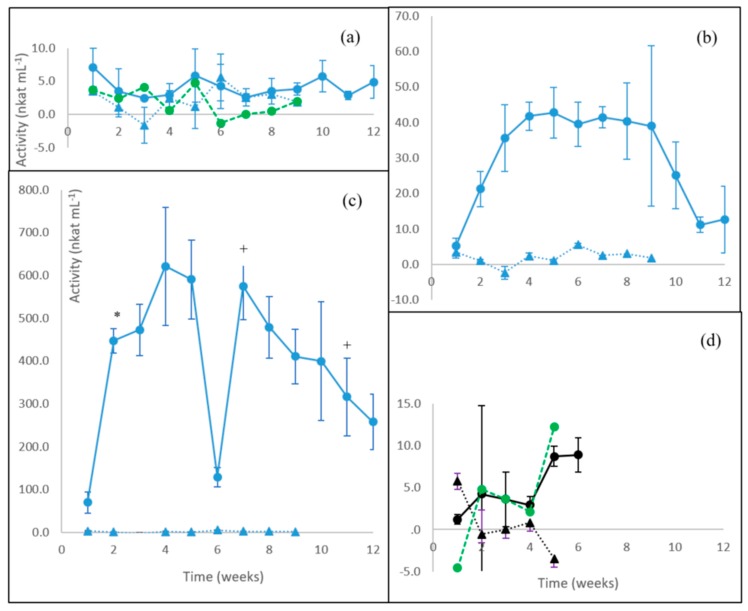
Xylanase activities produced by (**a**) the WR fungus *P. radiata*; (**b**) the BR fungus *F. pinicola*; (**c**) the GR fungus *S. commune*; and (**d**) the LDF species *C. cinerea*. Fungal cultivations on birch wood (blue line with spheres) and on barley straw (black line with spheres). Values from control incubations of birch wood (blue triangles) and barley straw (black triangles) without fungi are included. (**a**,**d**) Net activity values are also shown (green dotted lines) after subtracting the control activity. Mean value (*n* = 3) is presented at each week time point. Bars indicate the standard error. Notice different scales in the y axis. *, + statistically significant changes between time points. For information on the fungi, see Table 1.

Xylan cleaving activities were produced by the fungi in variable levels and profiles, especially on the lignocellulose substrates (Figure 5). The most fluctuating activity was observed on birch wood cultivations of *P. radiata* (Figure 5a). The net activity estimated (green line) slight low value peaks on cultivation weeks three and five (between 4 to 5 nkat mL^−1^), but due to the high standard deviation observed between the culture flasks, no further predictions on the enzyme production could be made.

The brown rot *F. pinicola* demonstrated an increasing trend of xylanase activity on birch wood until cultivation week four to a stable and lasting level of about 40 nkat mL^−1^ (Figure 5b). Between weeks 10 to 12, xylanase activity decreased to 12 nkat mL^−1^. However, the high deviation between the three culture flasks may distort the decline. With the intermediate “grey rot” fungus *S. commune*, however, even 10x higher production of xylanase activity was observed on birch wood reaching up to levels of up to 600 nkat mL^−1^ (Figure 5c). After a quick drop on the sixth cultivation week, xylanase activity quickly returned to the previous level, then declining to a level of about 10 nkat mL^−1^. The litter-decomposing fungus *C. cinerea* in turn demonstrated low levels of xylanase production on barley straw starting from week two reaching to a calculated value of 12 nkat mL^−1^ on week six (Figure 5d), thus resembling the slight xylanase production pattern seen with the white rot fungus *P. radiata* on birch wood (Figure 4a).

### 3.6. Summary of Enzyme Production in Fungal Cultures

A summary of the highest activities observed in the fungal cultures is presented in Table 2. Laccase and MnP activities observed for *P. radiata* were significantly higher (*p* ≤ 0.05) compared to the same activities observed for *F. pinicola* and *S. commune*, also growing on birch wood. Regarding β-glucosidase and xylanase activities, *S. commune* produced significantly higher activities (*p* ≤ 0.05) compared to *P. radiata* and *F. pinicola*. Endoglucanase activity ranged around 3 to 5 nkat mL^−1^ in all the fungi tested, and due to the constraints of the method, no further predictions could be made about which fungus produced the highest endoglucanase activity. In the case of *C. cinerea*, comparison with the other fungi was not performed since the fungus was growing on a different substrate (straw).

### 3.7. Extracellular pH and Concentration of Organic Acids

**Figure 6:**
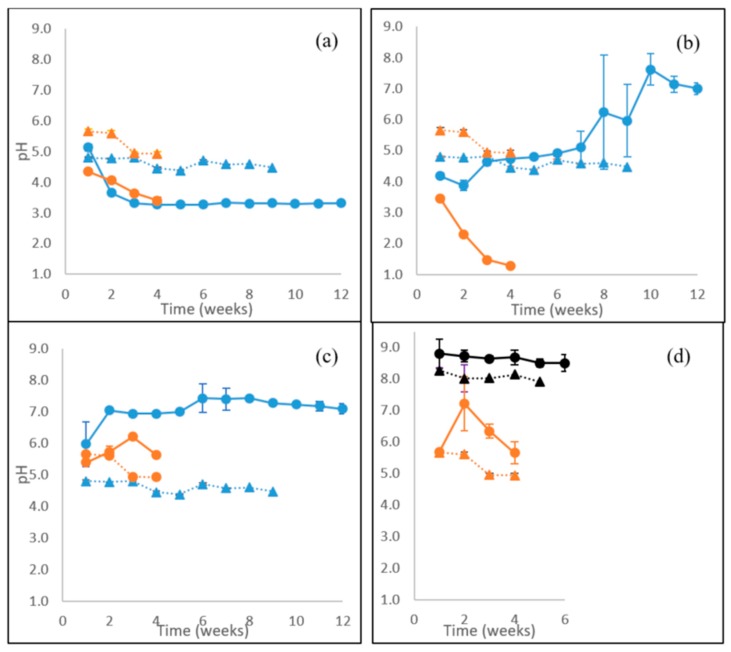
pH values recorded during 12 weeks of lignocellulose solid-state cultivations and 4 weeks on ME liquid medium, cultures of (**a**) WR fungus *P. radiata*; (**b**) BR fungus *F. pinicola*; (**c**) “GR” fungus *S. commune*; (**d**) LDF *C. cinerea*. Fungal cultivations were performed on birch wood (blue line with spheres) or barley straw (black line with spheres), and on liquid ME medium (orange line with spheres). Values from control incubations of birch wood (blue triangles, dotted line), barley straw (black triangles, dotted line) and liquid ME medium (orange triangles, dotted line) without fungi are included. Mean value (*n* = 3) is presented at each week time point. Bars indicate the standard error.

A variation in the pH values was observed in the course of cultivation time for *P. radiata*, *F. pinicola* and *S. commune* as solid-state cultures on birch wood and as well on the liquid ME medium (Figure 6a–c). As is seen in Figure 6, the water phase pH was almost constant (from pH 4.8 to pH 4.5) during 8 weeks of incubation of birch wood without fungi (control incubations). Acidity increased especially on birch wood with the WR fungus *P. radiata* from pH 5 in 2 weeks to pH 3.3, and thereby remaining constant until the end of cultivation (week twelve; Figure 6a). In contrast, the BR fungus *F. pinicola* caused a late increase in the pH value on birch wood up to a level of pH over 7 from week 10 until the end of cultivation (Figure 6b). On liquid ME medium, however, the BR fungus affected a rapid pH drop near to pH 1 in 4 weeks, whereas with *P. radiata*, a moderate drop to a level of pH 3.4 occurred (Figure 6a,b).

Similar to *F. pinicola*, an increment of alkalinity was observed on birch wood with the “GR” intermediate decay fungus *S. commune* (Figure 6c). On the second week of cultivation, pH increased from pH 6 to about pH 7 in 1 week, remaining at this level until the end of cultivation. In contrast to birch wood cultures, pH slightly increased in *S. commune* cultures on ME liquid medium to a level of pH 6. The ME medium stayed acidic dropping from pH 5.7 to pH 4.9 in 4 weeks (Figure 6). In the cultures of the LDF *C. cinerea* on barley straw, pH was over 8.5 during the 6 weeks of cultivation (Figure 6d). Notable is the high pH of the substrate barley straw (about pH 8). On the liquid ME medium, likewise an increase of pH occurred in cultures of *C. cinerea* up to pH 7.2 on week two, however, decreasing to below pH 6 within 2 weeks.

**Figure 7:**
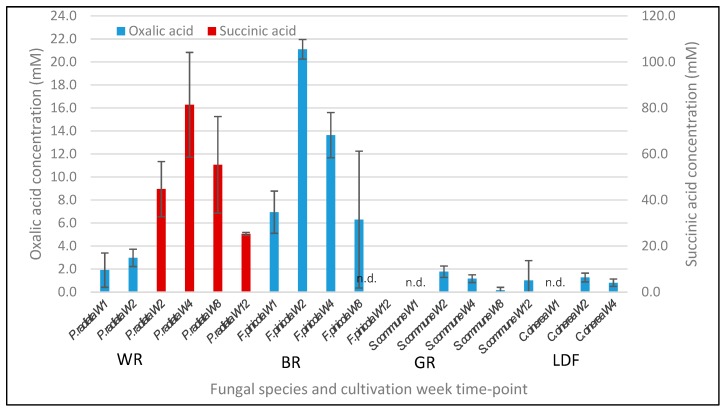
Accumulation of organic acids in the fungal solid-state cultures on lignocellulose substrates at weekly time points. Organic acids were measured by HPLC analysis of culture fluid samples. W1–W12; cultivation week 1–12. Concentration of fumaric acid in the cultures of *C. cinerea* on barley straw was lower than the detection limit (<0.5 mM) and thereby invisible in the figure. Mean value (*n* = 3) is presented at each week time point. Bars indicate the standard error. n.d., not detected; WR, white rot; BR, brown rot; GR, grey rot, intermediate decay type; LDF, litter-decomposing fungus.

Regarding the source of acidity changes in the solid-state cultures on lignocellulose substrates, concentrations of the fungal produced organic acids were determined by HPLC analysis of the culture fluids at chosen time-points: weeks 1, 2, 4, 8, and 12 for *P. radiata*, *F. pinicola* and *S. commune* and weeks 1, 2, 4, and 6 for *C. cinerea* (Figure 7). Notable are the high concentration levels of accumulation of oxalic acid in the birch wood cultures of the BR fungus *F. pinicola*, up to over 20 mM concentrations, and likewise high concentrations of predicted succinic acid (preliminary identification) in cultures of the WR fungus *P. radiata* after the second week of cultivation. In contrast to the WR and BR fungi, the “GR” fungus *S. commune* demonstrated weak production of oxalic acid below 2.0 mM on birch wood, and the LDF *C. cinerea* produced only ½ of this level on barley straw (Figure 7).

## 4. Discussion

Recent advances in accumulating genomic information by next-generation sequencing techniques have allowed targeted comparative genomic research on fungal genomes [4,6,8,9,10,11,12,18]. These wide studies have enhanced integration of genomic and genetic data to proteomic and metabolic analyses, and to knowledge on fungal ecology. Thereby, instead of the white rot–brown rot dichotomy, a new view of a continuum of various and maybe even fungal species-specific strategies for inhabitation of wood and decomposition of plant cell wall lignocelluloses is emerging [8,9,10]. One clear example is the *Agaricomycetes* species *Schizophyllum commune* of the order *Agaricales*, previously classified as a white rot fungus, despite the absence of lignin-modifying class-II peroxidases leading to disability to modify lignin [20,26]. In fact, *S. commune* may display a unique mechanism to decompose plant cell wall lignocelluloses including a combination of oxidative chemistry like observed with brown rot fungi, and efficient production of certain CAZymes. Thus, we aimed to explore the decomposition strategy of this fungus by analyzing its cellulolytic, hemicellulolytic and lignin-attacking CAZyme activities produced on wood substrate and in comparison with well-known fungi of white rot, brown rot, and litter-decomposing characteristics cultivated under similar laboratory conditions.

### 4.1. WR Fungus Enzyme Activity Profile

White rot fungi are well-known producers of oxidoreductases such as laccases and heme-including class-II peroxidases to attack lignin [1,2,3,4,5,6,12,14,15,16]. Accordingly, the white rot fungus *P. radiata* showed the highest laccase activities when cultivated on birch wood in our study. A similar pattern of an early-phase decay stage that produced laccase has been observed previously with *P. radiata*, and on spruce and coniferous wood, laccase activity was peaking within the first month of cultivation [12,22,24]. These observations confirm that the fungus is a strong producer of laccase on solid wood lignocellulose substrates. However, a higher level of laccase activity was now observed on birch wood than previously attained on coniferous wood [12,22,24,27], suggesting potential promotion of laccase production by the lignocellulose substrate. This effect may also be a consequence of more preferred growth on birch wood since the species has specificity of inhabitation and decomposition of dead deciduous wood in nature [28]. *P. radiata* also produced the highest MnP activities, and likewise observed previously on spruce wood, MnP production was occurring simultaneously with laccase activity [12,27,28]. On the carbohydrate-rich liquid ME medium, low laccase but distinct MnP activities were produced by *P. radiata*, demonstrating the stability of lignin-attacking oxidoreductase expression in the early phase of growth and decomposition processes.

White rot fungi are able to modify lignin and decompose wood cell wall polysaccharides leaving most of cellulose as a white residue [1,2,3,4,5]. Therefore, it was expected that the production of β-glucosidase activity by *P. radiata* would remain low during cultivations on birch wood, at the levels lower than 1 nkat mL^−1^ as was observed previously on spruce wood [24,27,28]. Regarding endoglucanase production, the enzyme activity profile was difficult to interpret due to interference in the assay method of a considerable accumulation of sugars released into the solution by fungal enzymatic activity, breaking wood cellulose and hemicelluloses into sugars [25,27]. However, some endoglucanase net activity could be observed after the third week on birch wood cultures of *P. radiata*, just after the peak of laccase and MnP activities. This indicates that cellulose degradation was promoted after the oxidoreductase enzyme production phase, similar to the timing and pattern of secreted enzyme production observed previously on spruce wood [12].

### 4.2. BR Fungus Enzyme Activity Profile

Even though three putative genes for laccase have been annotated in the genome of *F. pinicola* [4,10], no extracellular laccase activity was observed on birch wood with the fungus. A similar absence of laccase activity was observed in our previous study adopting the same isolate, both on semi-solid liquid cultures and solid-state cultures on coniferous wood [17,22,27]. These observations indicate that the presence of certain genes is not necessarily an indication of fungal expression of the enzyme and production of extracellular activity on natural, solid lignocellulose substrates. Considering class-II peroxidases for attack against lignin, this was excluded and MnP activity was not analyzed from *F. pinicola* cultures due to the absence of respective CAZy AA2 genes in the species genome [4,6,8,10].

It is considered that the breakdown of polysaccharides such as cellulose in brown rot fungi proceeds mainly non-enzymatically via Fenton chemistry reactions, involving some hydrolytic CAZyme activities as well [1,8,10,17]. In accordance, moderate endoglucanase and β-glucosidase enzyme activities were observed in this study for *F. pinicola* on birch wood. In previous studies, *F. pinicola* showed low cellulolytic enzyme activities on coniferous wood substrates [17,22,27], all suggesting that it is not a strong producer of cellulolytic enzymes. However, regarding the depolymerization of hemicellulose, *F. pinicola* demonstrated a high level of xylanase activity on birch wood in our study. Notable production of xylanase was evident with the same isolate on coniferous and spruce wood [22,27], and preference for hemicellulose breakdown has been reported for other species of *Polyporales* brown rot fungi [29,30,31]. These observations suggest that for *F. pinicola*, enzymatic attack against birch wood xylan is an important mechanism to release hemicellulose sugars together with non-enzymatic decomposition reactions against cellulose.

### 4.3. Intermediate “GR” Fungus Enzyme Activity Production Profile

A preference for decomposing wood polysaccharides has been predicted for the intermediate rot fungus *S. commune* due to the identification of divergent CAZy genes in its genome, especially extensive sets encoding cellulolytic and hemicellulolytic activities [4,8,11,20,29,32]. For instance, in cultivations using Jerusalem artichoke as a growth substrate, *S. commune* produced high cellulolytic activity compared to white rot fungi (*Phanerochaete chrysosporium* and *Ceriporiopsis subvermispora*) and the brown rot fungus *Gloeophyllum trabeum* [26]. In accordance with these findings, *S. commune* was a strong producer of cellulolytic β-glucosidase on birch wood in this study, reaching over 10 times higher activity values than observed for the white rot and brown rot fungi (*P. radiata* and *F. pinicola*, respectively) on the same substrate. However, production of the cellulolytic endoglucanase was not reaching such activity levels as observed for β-glucosidase on birch wood with *S. commune*. These observations suggest a notable role for β-glucosidases in *S. commune* for growth on birch wood.

Regarding hemicellulose degradation, *S. commune* showed remarkably high production levels of xylanase activity in comparison to the white rot and brown rot fungi, and the second *Agaricales* species, the litter-decomposing fungus *C. cinerea* cultivated on barley straw. High extracellular xylanase activities produced by *S. commune* have been observed on plant biomass-based solid-state cultures previously, with even higher activity values than measured in a commercial enzyme cocktail of the *Ascomycota* fungus *Trichoderma longibranchiatum* [26]. In our study, xylanase activity on birch wood was observed in two cycles, which may be explained by differential regulation of multiple xylanase-encoding CAZy genes during the cultivation time. Overall, these findings imply that *S. commune* has an efficient machinery for hemicellulose breakdown.

Similar to the brown rot fungus *F. pinicola*, *S. commune* genome lacks the genes coding for lignin-modifying class-II peroxidases, thus confirming inability of the fungus to decompose wood lignin [4,8,20,26]. Accordingly, no MnP activity was observed in *S. commune* cultures on birch wood or on the liquid ME medium. A second similarity in the deficiency of production of lignin-modifying enzyme activities between *S. commune* and *F. pinicola* was observed: No laccase activity was detected on birch wood for *S. commune* even though two laccase genes have been identified in its genome [4,20]. This may be explained again that laccase-encoding genes were not expressed on birch wood under the cultivation conditions of this study.

### 4.4. LDF Enzyme Activity Production Profile

Seventeen laccase-encoding genes of two subfamilies are annotated and cloned from *C. cinerea* [33]. Accordingly, it was expected to observe notable extracellular laccase production on the solid-state cultures of *C. cinerea* on barley straw. However, only transient laccase activity on the second week was detectable in our cultivations. In other studies performed with *C. cinerea* using rice straw and paragrass as solid lignocellulose substrates [34,35], moderate laccase activities were observed after 2 weeks of cultivation, which implies some role for extracellular laccase in the growth and colonization of solid plant biomass substrates for *C. cinerea*. However, considering the role of laccases in filamentous fungi including involvement in various physiological processes such as hyphal growth and fusion, fruiting body formation and sporulation, it is more reasonable that expression of the multiple laccases is more regulated by fungal life cycle than the growth substrate [15,33]. In this study, the formation of fruiting bodies (basidiocarps) of *C. cinerea* was already observed on the second week on barley straw, with continuous growth and development until the end of cultivation. This indicates that the fungal mycelium was not secreting laccase enzyme efficiently and thereby, laccases of *C. cinerea* were less involved in decomposition of the barley straw substrate.

*C. cinerea* produces its own type of extracellular class-II peroxidase (CiP), which is a low-redox potential enzyme, and thus, is inefficient in oxidation of lignin units [4,14]. Therefore, detected MnP activity on barley straw cultures of *C. cinerea* was most likely caused by moderate production of CiP during the cultivation time. Regarding cellulolytic activity of *C. cinerea*, low levels of both β-glucosidase and endoglucanase activities were produced (4 nkat mL^−1^), which is somewhat less than the β-glucosidase activity observed previously using rice straw as a substrate [34]. On the other hand, xylanase was the highest enzyme activity observed on barley straw with *C. cinerea*, especially during the last 2 weeks of cultivation. This pattern of xylanase enzyme activity was similar to results attained with *C. cinerea* on paragrass [35]. In summary of our observations on enzyme activities produced in the solid-state cultures, *C. cinerea* seemingly preferred degradation of hemicellulose rather than cellulose of barley straw.

### 4.5. Extracellular pH and Production of Organic Acids

Filamentous fungi secrete organic acids for several physiological purposes, that is to keep a favorable environment for enzymatic and biochemical decomposition of lignocellulose components, for extracellular acidity-promoting hyphal growth against competing microbes; promote oxidative Fenton reactions; chelate and dissolve nutritional cations; aid lignin modification and decomposition by chelating Mn^3+^ ions; and produce extracellular H_2_O_2_ and other reactive oxygen species [1,10,16,36,37].

As expected, the brown rot fungus *F. pinicola* produced high concentrations (over 20 mM) of oxalic acid on birch wood, which is in accordance with our previous observations with the fungus cultivated on spruce and coniferous wood and in liquid media [17,22,27]. Strong production of extracellular oxalic acid by brown rot fungi, leading to a rapid decline of pH even to levels lower than pH 1, is thought to be involved in the maintenance of a proper environment for the Fenton reactions [10,16,17,27]. An explanation for the decline in oxalic acid concentration together with an observed increase in the pH value in the liquid phase of the birch wood cultures of *F. pinicola* is that the secreted oxalic acid dissociates to oxalate anions, which in turn attach to solid lignocellulose wood particles. Thereby, lower amounts of free oxalic acid are detectable in the liquid phase, as was observed recently for the fungus when cultivated on spruce wood [27].

With the white rot fungus *P. radiata*, low production of oxalic acid has been observed on spruce wood [27]. However, in our current study on birch wood as a substrate, acidification of the culture liquid phase to pH 3 was observed in 3 weeks, which is apparently due to the accumulation of notable amounts of succinic acid by *P. radiata*. One explanation for this finding is that secreted succinic acid may have served as an extracellular reserve for the mycelium for cellular redox and electron transfer shuttling together with enhancement of decomposition of the wood substrate. However, succinic acid was not detected with the other three fungi cultivated on solid lignocelluloses, and our annotation of the produced acidic organic compound is only preliminary. With the two *Agaricales* fungi studied, *S. commune* and *C. cinerea*, only low amounts of extracellular oxalic acid were observable on their solid lignocellulose substrates, which is in accordance with the high pH levels generated in the cultures (over pH 7 for *S. commune* on birch wood, over pH 9 for *C. cinerea* on barley straw).

## 5. Conclusions

Overall, the results of this study confirm that the enzyme activities observed on birch wood with *P. radiata* followed an expected pattern of white rot fungal decomposition strategy, initiated with the production of high laccase and MnP activities, and were similar to the observations of previous studies performed on wood substrates with the same fungus. Furthermore, the high amounts of succinic acid accumulating in the aqueous phase of *P. radiata* cultivations on birch wood may indicate an influence of the substrate in regulation of CAZYme expression and a possible induction of metabolic routes not previously observed on other lignocellulosic substrates. Considering the brown rot fungus *F. pinicola*, its notable that the production of xylanase activity on birch wood contrasted with moderate cellulolytic (β-glucosidase and endoglucanase) activities produced on the same substrate. This suggests the primary effect of the oxidative Fenton reactions as the main mechanism of *F. pinicola* in the decomposition of wood cellulose. The intermediate “grey rot” fungus *S. commune* in turn demonstrated strong production of β-glucosidase, and especially, remarkable levels of xylanase activity on birch wood substrate. These findings point to great potential of *S. commune* in decomposition of hemicelluloses, and furthermore, potentiality in biotechnological applications on lignocellulose bioconversion and pretreatment. Likewise, the litter-decomposer *C. cinerea* apparently had a preference on hemicellulose decomposition on the barley straw substrate, with negligible lignin-modifying and cellulolytic activities observed in 6 weeks of cultivation. Thus, regarding the production of xylanase and degradation of hemicellulose in wood and grass plant biomasses, the *Agaricales* species *S. commune* and *C. cinerea*, respectively, show high potentiality. Our findings indicate that the two *Agaricales* fungi have a very different enzyme mechanism for decomposition of lignocellulose from the strategy of wood white rot or brown rot fungi, which is also independent of the production of extracellular acidity by secretion of organic acids like oxalate.

## Figures and Tables

**Table 1:** Fungal isolates used in this study.

Fungal Species	Isolate Code	Decay Strategy ^1^	Preferred Substrate ^2^	Reference
*Phlebia radiata*	FBCC0043	WR	D wood	[12,22]
*Fomitopsis pinicola*	FBCC1181	BR	D&C wood	[17,22]
*Schizophyllum commune*	H4-8	“GR”	D wood	[20]
*Coprinopsis cinerea*	*AmutBmut* P^+^B^−^	LDF	Grass plant LC	[21]

^1^ WR, white rot; BR, brown rot; “GR”, intermediate grey rot; LDF, litter-decomposing fungus. ^2^ D, deciduous; C, coniferous; LC, lignocellulose.

**Table 2:** Highest enzyme activities (nkat mL^−1^) measured in the solid-state lignocellulose cultivations on birch wood or barley straw. Mean values of three biological replicate cultures with standard deviation are presented.

Highest Enzyme Activity (nkat mL^−1^)	Week	WR *Phlebia radiata*	Week	BR *Fomitopsis pinicola*	Week	“GR” *Schizophyllum commune*	Week	LDF * Coprinopsis cinerea
Laccase	3	13.9 ± 2.7	10	0 ^1^	6	0 ^1^	2	5.5 ± 7.8
MnP	2	2.4 + 0.0	NA ^2^	NA ^2^	11	0 ^1^	5	2.9 ± 2.5
β-glucosidase	1	0.6 ± 0.2	9	1.5 ± 1.1	4	53.6 ± 3.5	3	2.0 ± 0.4
Endoglucase	2	3.6 ± 0.4	3	5.5 ± 1.0	5	4.4 ± 1.3	5	3.2 ± 0.8
Xylanase	1	7.0 ± 2.9	5	42.8 ± 7.1	4	621 ± 138	6	8.9 ± 2.0

^1^ 0 value, under detection limit; ^2^ NA, non-assayed; * on barley straw.
